# *PLOD3* suppression exerts an anti-tumor effect on human lung cancer cells by modulating the PKC-delta signaling pathway

**DOI:** 10.1038/s41419-019-1405-8

**Published:** 2019-02-15

**Authors:** Jeong-Hwa Baek, Hong Shik Yun, Gyoo Taik Kwon, Janet Lee, Ju-Young Kim, Yunhui Jo, Jae-Min Cho, Chang-Woo Lee, Jie-Young Song, Jiyeon Ahn, Jae-Sung Kim, Eun Ho Kim, Sang-Gu Hwang

**Affiliations:** 1 Division of Radiation Biomedical Research, Korea Institute of Radiological & Medical Sciences, Seoul, 01812 Korea; 2 Department of Molecular Cell Biology, Sungkyunkwan University School of Medicine, Suwon, 440-746 Korea

## Abstract

Current lung cancer treatments are far from satisfactory; thus, finding novel treatment targets is crucial. We recently identified procollagen-lysine, 2-oxoglutarate 5-dioxygenase 3 (PLOD3), which is involved in fibrosis and tissue remodeling as a radioresistance-related protein in lung cancer cells; however, its mechanism is unclear. In this study, we designed human *PLOD3*-specific short interfering (si)RNAs and tested their effects on tumor growth inhibition in vitro and in vivo. *PLOD3* knockdown overcame chemoresistance and decreased radioresistance by inducing caspase-3-dependent apoptosis in lung cancer cells. Furthermore, PLOD3 interacted with PKCδ to activate caspase-2,4-dependent apoptosis through ER-stress-induced IRE1α activation and the downstream unfolded-protein response pathway. In a mouse xenograft model, *PLOD3* knockdown promoted radiation-induced tumor growth inhibition, without side effects. Moreover, lung cancer patients with high PLOD3 expression showed poorer prognosis than those with low PLOD3 expression upon radiotherapy, suggesting that PLOD3 promotes tumor growth. Therefore, *PLOD3* siRNA suppresses radioresistance and chemoresistance by inducing apoptosis and renders PLOD3 as a candidate lung cancer biomarker. *PLOD3* gene therapy might enhance the efficacy of radiotherapy or chemotherapy in lung cancer patients.

## Introduction

Lung cancer is the main cause of cancer-related morbidity, and non-small-cell lung cancer accounts for 80–85% of all lung cancer cases^1^. However, among these patients, only 10% achieve a complete response, and the total 5-year survival rate has remained dismal at 15%^2^ because radiation resistance severely affects the efficacy of radiotherapy^3,4^. Thus, we highlight the need for a greater understanding of the cellular and molecular targets that drive tumorigenesis to achieve better treatment efficacies.

Recently, we found four proteins, including procollagen-lysine, 2-oxoglutarate 5-dioxygenase 3 (PLOD3), which had not been previously reported to be related to radioresistance or chemoresistance^5^. PLOD proteins, are involved in fibrotic processes and tissue remodeling^6,7^. Three highly homologous PLOD isoforms have been characterized to date, including PLOD2, and PLOD3^8^. *PLOD3* is localized on chromosome 7q36^9^, and PLOD3 activity is critical for the biosynthesis of type IV and VI collagens^10^. Mutations in human *PLOD3* result in congenital disorders that influence the connective tissues of various organs^11^, suggesting that PLOD3 is crucial for normal collagen function. Collagen also is involved in tumor progression by modulating cancer cell migration, invasion^12^, proliferation^13^, survival^14^, and metastasis^15^. Based on these facts, we focused on cancer cell survival with respect to PLOD3 function.

Two independent studies have reported *PLOD3* mRNA overexpression in glioma and hepatocellular carcinoma tissues^16–18^. *PLOD3* overexpression was correlated with higher circulating protein levels in some patients^19^. However, the molecular mechanisms underlying the role of PLOD3 in lung cancer cell death have not been fully elucidated, and there are no data regarding the possible role of PLOD3 in lung cancer cell apoptosis. Further, the oncogenic function and prognostic value of this protein as a therapeutic and diagnostic target for lung cancer have not been revealed.

We previously found that the mechanistic target of PLOD3-induced cell death is the endoplasmic reticulum (ER)-associated stress-induced apoptosis pathway^20,21^, which, under physiological conditions, is activated by the accumulation of misfolded proteins in the ER to maintain cell survival^22^. Specifically, ER stress leads to the activation of three major unfolded protein response sensors, including pancreatic eIF2-α kinase (PERK), high inositol-requiring 1 (IRE1-α), and ATF6. First, PERK phosphorylates the eukaryotic translation initiation factor-2a, resulting in both an initial decrease in general translation initiation and the selective translation of the transcription factor ATF6. Second, ATF6 induces growth arrest and DNA damage-inducible proteins (GADD153/CHOP), leading to cell-cycle arrest, hence preventing the damage to the cell^23,24^. IRE1-α mediates the splicing of X-box-binding protein 1, which increases the transcription of ER-resident chaperones, folding enzymes, and components of the protein degradation machinery. Third, ATF6, after activating cleavage, results in both the induction of CHOP and the upregulation of protein folding and degradation^24^. Prolonged, unresolvable ER stress overrides the salvage mechanisms of the initial unfolded protein response and eventually leads to apoptosis involving CHOP signaling, JNK activation, bcl-2 phosphorylation and depletion, and caspase cleavage (e.g., caspase-4).

Protein kinase C (PKC) isozymes comprise a family of at least 10 related serine-threonine kinases that play critical roles in the regulation of several cellular processes, including proliferation, cell-cycle regulation, differentiation, malignant transformation, and apoptosis^25^. Based on their structures and cofactor requirements, PKC isoforms are divided into classic PKC (α, β1, β2, and γ), novel (δ, ϵ, η, and θ), and atypical (ζ and λ/i) groups^25^. Members of this family are either pro-apoptotic or anti-apoptotic, depending on the isoform and cellular context. For example, PKCα and PKCϵ inhibit apoptosis by phosphorylating or increasing the expression of the anti-apoptotic protein Bcl-2, whereas the caspase-3-dependent and caspase-2-dependent activation of PKCδ promotes apoptosis via tyrosine phosphorylation, association with specific apoptotic proteins, and translocation of activated PKCδ to the mitochondria^26^.

Here, to develop an anti-tumor reagent, we designed human *PLOD3*-specific short interfering (si)RNAs to knockdown the endogenous PLOD3 overexpression in lung cancer and then investigate cell proliferation and cell death. We also assessed the possible involvement of PKCδ and PLOD3 in the ER-stress-induced cell death pathway in cell culture and in an in vivo model.

## Results

### PLOD3 downregulation decreases radioresistance in R-H460 cells

**Fig. 1:**
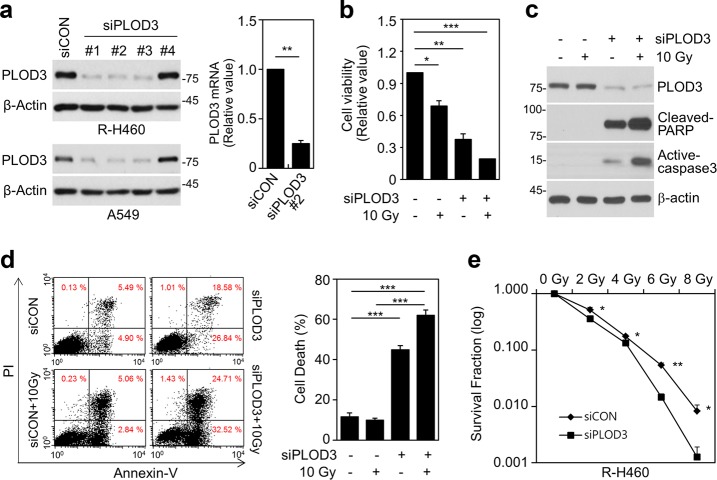
Downregulation of PLOD3 decreases radioresistance in R-H460 cells. **a** R-H460 cells were transfected with 40 nM control siRNA (siCON) or *PLOD3* siRNA (siPLOD3) for 24 h. Transcript and protein levels of PLOD3 were determined by western blotting (left) and qRT-PCR (right). **P < 0.01. **b** Analysis of the viability of cells treated with or without 10 Gy radiation after transfection with siCON or siPLOD3. *P < 0.05; **P < 0.01; ***P < 0.001. **c** Protein levels of PLOD3, cleaved PARP, and active caspase-3 (cell-death marker) as determined by western blotting. **d** Determination of cell death in R-H460 cells (treated as in **b**) by AV/PI staining. ***P < 0.001. **e** Surviving fraction of cells treated with a single dose of radiation (0–8 Gy) after transfection with siCON or siPLOD3, measured two weeks after radiation treatment. *P < 0.05; **P < 0.01

To determine whether PLOD3 is involved in regulating radioresistance, we tested the effects of PLOD3 depletion on cell viability and death (Fig. 1a). After siRNA transfection for 48 h, cell viability was significantly decreased in cells transfected with *PLOD3* siRNA compared to that in control siRNA-transfected cells, and this decrease was enhanced in irradiated cells (Fig. 1b, Supplementary Figure 1a). We next confirmed that PLOD3 knockdown alone or in combination with radiation led to upregulation of cleaved PARP and active-caspase-3 levels (Fig. 1c, Supplementary Figure 1b). FACS analysis showed that PLOD3 knockdown in A549 cells increased cell death (~45%) by more than 3.8-fold compared to that in control cells (~12%), and 10-Gy radiation further increased cell death slightly (Supplementary Figure 1c, d). However, in R-H469 and A549 cells, the combination of PLOD3 knockdown and 10-Gy radiation significantly increased cell death compared to that in control cells (Fig. 1d, Supplementary Figure 1c, d). Additionally, the number of colonies formed decreased with increasing radiation doses in *PLOD3* siRNA-transfected R-H460 and A549 cells, indicating a dose-dependent relationship (Fig. 1e, Supplementary Fig 1e). Therefore, our results suggest that PLOD3 depletion induces cell death to regulate radiosensitivity.

### PLOD3 downregulation promotes radiosensitivity and inhibits tumorigenesis in vivo

**Fig. 2:**
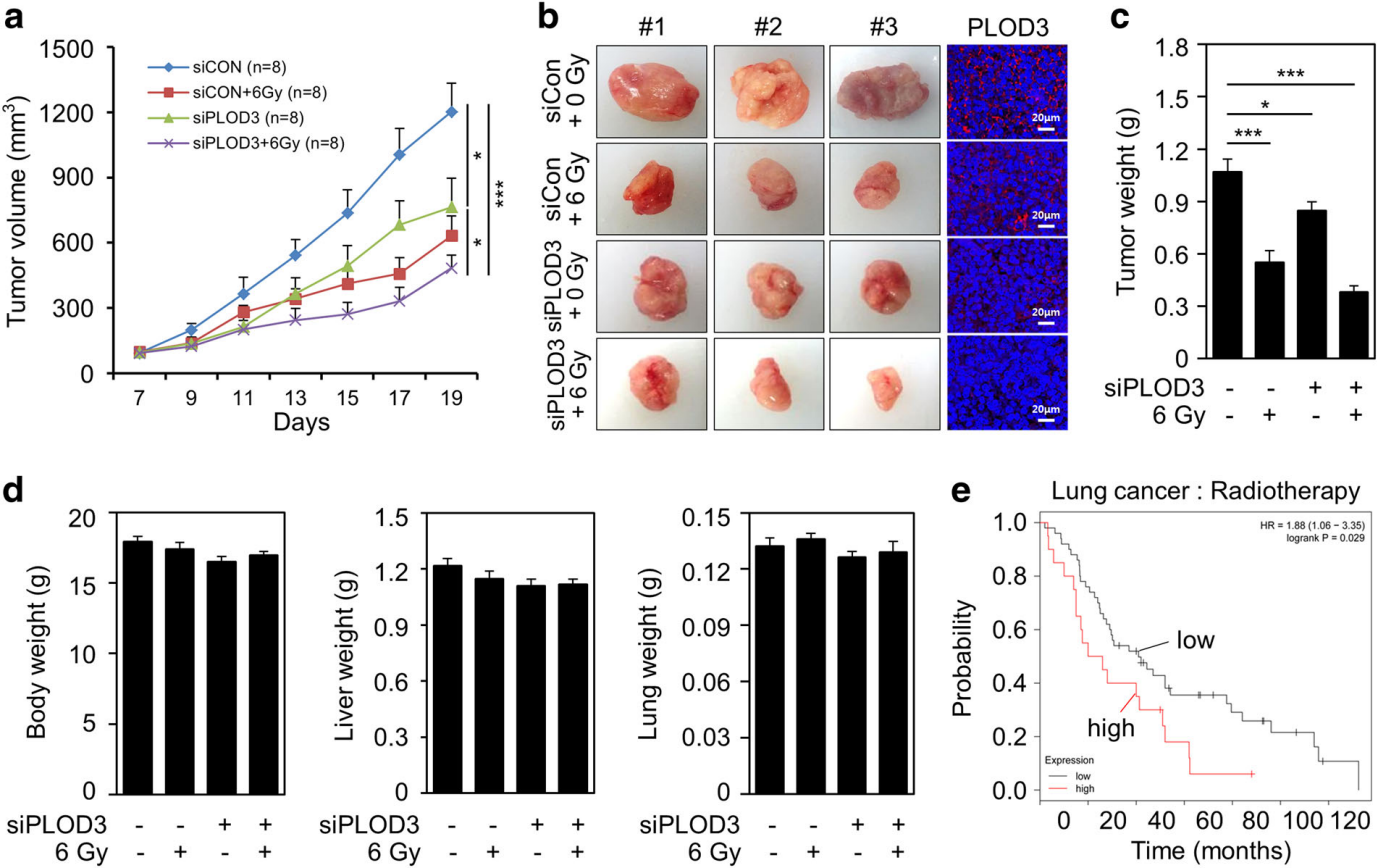
Reduced PLOD3 suppresses radioresistance in R-H460 cell xenograft tumor model. **a** Tumor volume was calculated at the indicated times using the formula: volume = (length × width^2^ × 3.14)/6; (*n* = 8). *P < 0.05; ***P < 0.001. **b** Tumors were excised and weighed at the end of the experiment (14 days). Representative images of tumors and tumor slides subjected to immunohistochemistry using an anti-PLOD3 antibody. **c** Effects of siRNA and radiation on tumor weights. Data shown are the means of each group. *P < 0.05; ***P < 0.001. **d** Body, liver, and lung tissues of mice were excised and weighed at the end of the experiment. All data are shown as the mean ± SD. **e** Five-year survival rate curves were obtained using Kaplan–Meier Plotter (www.kmplot.com) and display the survival probability based on data of 68 lung cancer patients treated with radiotherapy

After confirming PLOD3 transfection efficiency in vivo, we analyzed the synergistic effect of radiation and *PLOD3* siRNA on R-H460 xenograft tumors. As indicated in Fig. 2a and Supplementary Fig. 2a, tumor volumes were markedly decreased by more than ~36% and 47%, compared to those in the control siRNA group, in the *PLOD3* siRNA-treated group and radiation-treated group, respectively. The most pronounced tumor growth-inhibitory effect (60%) was observed in the *PLOD3* siRNA plus irradiation group (Fig. 2b). Further, PLOD3 protein expression was diminished in *PLOD3* siRNA-injected mice compared to that in control siRNA-treated mice (Fig. 2b). Tumor weight measurements were in agreement with tumor volume results (Fig. 2c). Moreover, the *PLOD3* siRNA plus radiation group showed further decreased PLOD3 protein levels compared to those in mice treated with either alone. There were possibly no visible signs of toxicity due to *PLOD3* siRNA and radiation in mice, as shown by the lack of differences in body, liver, and lung weights (Fig. 2d).

Patients with high PLOD3-expressing lung cancer who were treated with radiotherapy had poorer prognosis than patients with low PLOD3-expressing lung cancer who were treated with radiotherapy in the Kaplan–Meier Plotter database (Fig. 2e). Collectively, these findings suggest that PLOD3 is correlated with tumor growth.

### PLOD3 knockdown induces caspase-dependent apoptosis in R-H460 cells

**Fig. 3:**
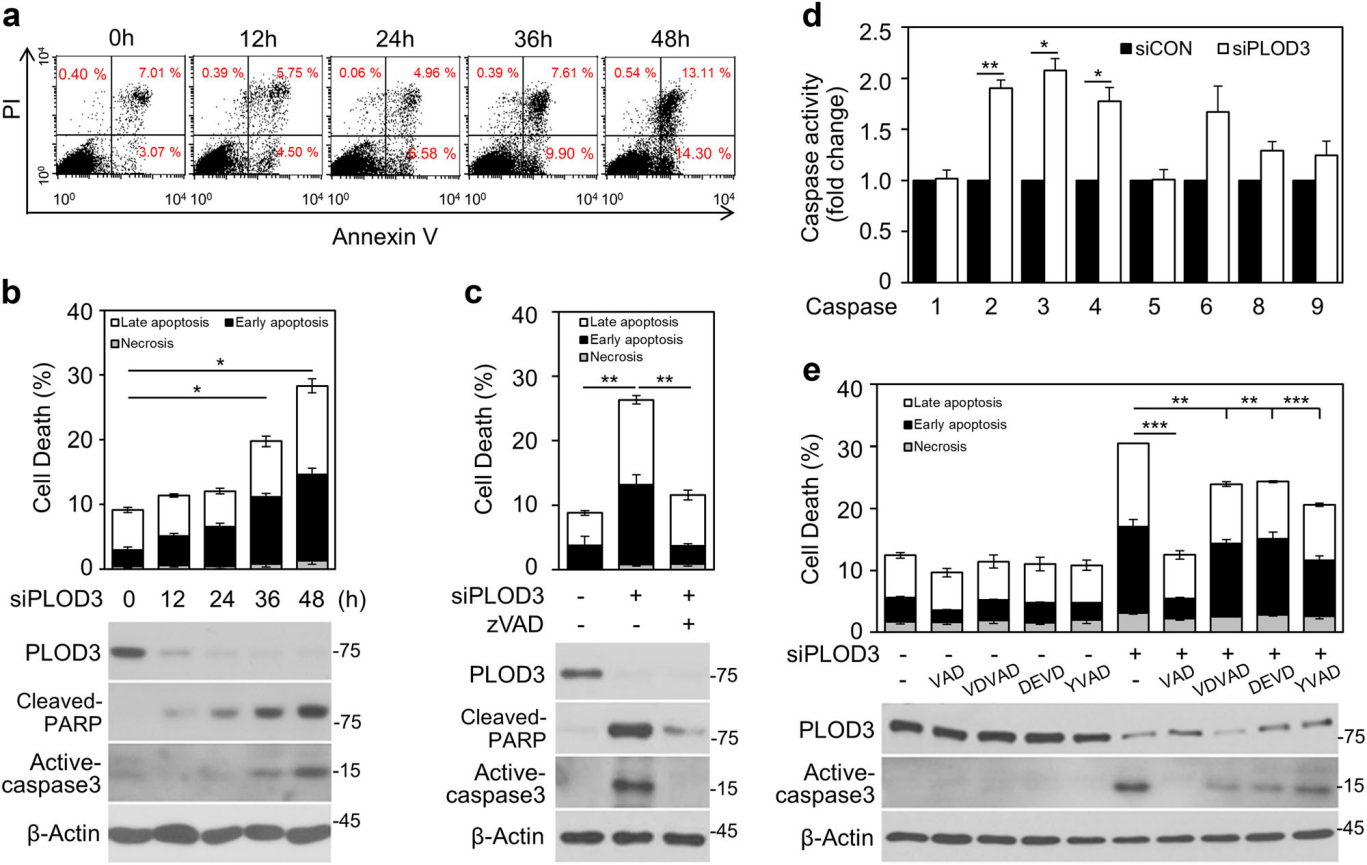
PLOD3 knockdown induces caspase-dependent apoptosis in R-H460 cells. **a**, **b** Cell death in cells transfected with 40 nM siPLOD3 as determined by AV/PI staining and western blotting at 12-h intervals. *P < 0.05. **c** R-H460 cells were treated with or without 40 µM Z-VAD-FMK (a pan-caspase inhibitor) after transfection with *PLOD3* siRNA. Cell death was measured 48 h after treatment by AV/PI staining and western blotting. **P < 0.01. **d** Analysis of caspase activity in R-H460 cells 48 h after transfection with *PLOD3* siRNA by ELISA. Data were collected using a Multiskan EX at 405 nm. *P < 0.05; **P < 0.01. **e** R-H460 cells were treated with Z-VAD-FMK (40 µM), Z-VDVAD-FMK (40 µM), Z-DEVD-FMK (40 µM), or Z-YVAD-FMK (40 µM) and then subjected to AV/PI staining and western blotting for active-caspase-3. **P < 0.01; ***P < 0.001

To investigate the mechanisms associated with PLOD3 knockdown-induced cell death, we assessed apoptosis and necrosis in R-H460 cells by flow cytometry (Fig. 3a). At 36 h, PLOD3 *siRNA* significantly increased apoptosis (AV-positive) by more than 2-fold (approximately 20%) compared to control siRNA. Further, *PLOD3* siRNA-mediated apoptosis was accelerated as incubation time increased, but necrosis (AV-negative/PI-positive) was not triggered (Fig. 3b). Caspase-3 cleavage was observed at the same time point as increased apoptosis by immunoblotting analysis. Moreover, *PLOD3* siRNA-induced apoptosis was caspase-dependent because Z-VAD-FMK, a pan-caspase inhibitor, significantly lowered the percentage of AV-positive cells and blocked the cleavage of caspase-3 (Fig. 3c). In A549 cells, FACS and western blot analysis revealed that Z-VAD-FMK treatment decreased PLOD3 depletion-induced apoptotic cell death (Supplementary Figure 3a, b). To investigate whether cleaved caspase-3 modulates caspase activation in PLOD3-depleted cells, we measured the activity of caspase family members in R-H460 cells (Fig. 3d). *PLOD3* siRNA significantly increased caspase-2, 3, 4, and 6 activity by ~1.9-, 2.1-, 1.8-, and 1.7-fold, respectively, compared to control siRNA. Accordingly, to investigate whether caspase inhibitors could block PLOD3 knockdown-induced apoptosis (Fig. 3e), R-H460 cells were treated with these compounds for 1 h and then transfected with *PLOD3* siRNA. Based on AV/PI double staining, Z-VAD-FMK, Z-VDVAD-FMK, Z-DEVD-FMK, and Z-YVAD-FMK treatment reduced apoptosis by approximately 18%, 7%, 6%, and 10%, respectively, in PLOD3-knockdown cells. Immunoblotting results confirmed that active caspase-3 was remarkably decreased by Z-VAD-FMK treatment, without rescuing PLOD3 protein levels, but was only slightly reduced by Z-VDVAD-FMK, Z-DEVD-FMK, and Z-YVAD-FMK. To identify additional possible mechanisms of cellular death, we investigated autophagy and proteasome activity, which are among other mechanisms associated with anti-cancer activity. We first treated cells with *PLOD3* siRNA to examine chymotrypsin-, caspase- and trypsin-like activities in R-H460 cells. Only minor changes were observed in the levels of the three proteasomal activities after *PLOD3* siRNA treatment (Supplementary Figure 3c). After treatment with 3-MA, which is widely used to inhibit autophagy, R-H460 cells were not significantly rescued from cell death induced by *PLOD3* siRNA, and morphological changes throughout the cytoplasm and in the cell membrane were not observed (Supplementary Fig. 3d). Thus, PLOD3 ablation induces cell death that depends on apoptosis, but not autophagy and proteasome activity.

### PLOD3 depletion induces DNA damage and ER stress-dependent caspase activation in R-H460 cells

**Fig. 4:**
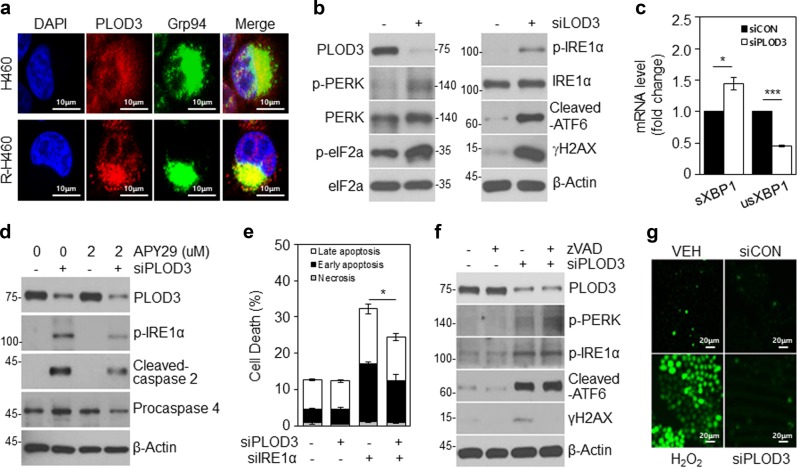
PLOD3 knockdown induces DNA damage and ER stress-dependent caspase activation in R-H460 cells. **a** Cells were fixed with 4% paraformaldehyde and immunostained using antibodies targeting PLOD3 and Grp94 (ER marker). DNA was visualized by DAPI staining. **b** At 24-h post-transfection with siCON or siPLOD3, cell lysates were prepared and used for immunoblotting with antibodies against ER stress markers (ATF6, PERK, and IRE1α) and a DNA damage marker (γH2AX). **c** qRT-PCR analysis for IRE1α activation, spliced XBP-1 (sXBP-1), and unspliced X-box-binding protein 1 (unXBP-1). The *GAPDH* mRNA was utilized for normalization. *P < 0.05; ***P < 0.001. **d** R-H460 cells were pre-treated with APY29 (IRE1α inhibitor), and after transfection with siPLOD3, cell lysates were prepared and used for immunoblotting with antibodies against p-IRE1α, caspase-2, and caspase-4. **e** AV/PI staining of cells transfected with siCON or siPLOD3 and/or siIRE1α for 48 h. *P < 0.05. **f** R-H460 cells were treated with or without 40 µM Z-VAD-FMK after transfection with siPLOD3, and cell lysates were used for immunoblotting with antibodies against ATF6, p-PERK, p-IRE1α, and γH2AX. **g** R-H460 cells were transfected with 40 nM siCON or siPLOD3 for 36 h and then stained with 2′,7'-dichlorofluorescein diacetate. Intracellular levels of reactive oxygen species were observed by confocal microscopy

Caspase-2 has been shown to mediate DNA damage-induced apoptosis, reactive oxygen species (ROS) accumulation, and ER stress^27^. Caspase-4 is also activated by ER stress^28^. First, to investigate the ER localization of PLOD3 in H460 and R-H460 cells, following treatments, these organelles were visualized using Grp94 as an ER stress sensor protein, and nuclei were stained with DAPI. PLOD3 was distributed homogeneously in the cytoplasm in co-localization with Grp94 (Fig. 4a). Immunoblotting revealed that PLOD3 depletion resulted in elevated cleaved ATF6 and phosphorylation of PERK and IRE1α as ER stress sensor proteins and also upregulated the DNA damage marker, γH2AX (Fig. 4b). qPCR confirmed increased splicing of *XBP-1*, a downstream target of IRE1α (Fig. 4c). Together with ER stress and the activation of the unfolded protein response system, IRE1α is reportedly involved in caspase activation. Thus, we examined whether IRE1α inhibition blocked caspase activation and cell death in PLOD3-depleted cells. The IRE1α inhibitor APY29 reduced PLOD3 knockdown-induced phosphorylation of IRE1α. APY29 also reduced cleaved caspase-2 and procaspase-4 and the proportion of apoptotic cells (Fig. 4d). R-H460 cells were treated with combinations of *PLOD3*, *IRE1α*, and control siRNA for 48 h and apoptosis was analyzed by AV/PI staining (Fig. 4e). *PLOD3* siRNA led to an increase in the apoptotic cell population (~32%); however, combined transfection with *IRE1α* siRNA significantly reduced this by more than 8% compared to *PLOD3* siRNA alone. Notably, we found that caspase inhibition in PLOD3-knockdown R-H460 cells did not regulate ER stress (Fig. 4f). Furthermore, caspase inhibition using Z-VAD-FMK completely inhibited γH2AX accumulation after PLOD3 knockdown (Fig. 4f). These results suggested that DNA damage is induced by caspase activity after PLOD3 depletion. However, PLOD3 knockdown-induced cell death was not found to depend on ROS levels (Fig. 4g). These results suggest that caspase-2 and 4 depend on IRE1α activation in *PLOD3* siRNA-treated cells.

### PLOD3 regulates PKC-dependent apoptosis by activating PKCα and PKCδ via direct interactions

**Fig. 5:**
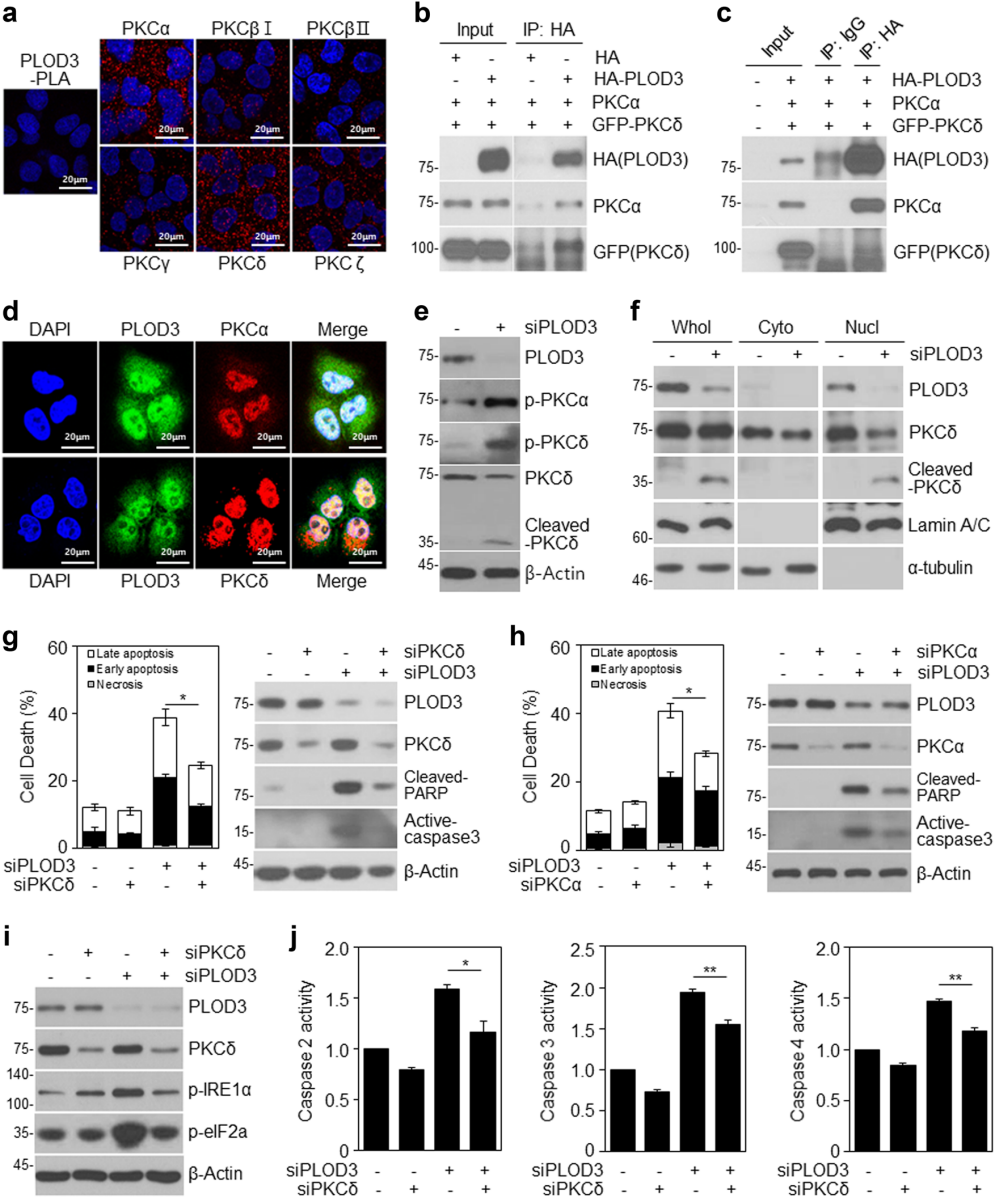
PLOD3 knockdown induces activation of PKCs and PLOD3 directly interacts with PKCs. **a** R-H460 cells were fixed and incubated with a mouse anti-PLOD3 antibody together with rabbit antibodies against PKCs, followed by in situ proximity ligation assay analysis. Representative confocal images of cells with proximity ligation assay-positive signals (red dots). **b**, **c** R-H460 cells were transfected with HA-PLOD3, PKCα, or GFP-PKCδ. Cell lysates were immunoprecipitated with normal IgG (a negative control) or HA antibody, and then immunocomplexes were resolved by SDS-PAGE and immunoblotted with antibodies against HA, PKCα, and GFP. **d** Immunostaining analysis of the localization of PKCs in R-H460 cells. Cells were fixed with 4% paraformaldehyde and immunostained for PLOD3 (green), PKCα (red), and PKCδ (red). DNA was visualized by DAPI staining. **e** *PLOD3* or control siRNA was transfected into R-H460 cells; 24-h post-transfection, cell lysates were prepared and used in immunoblotting with antibodies targeting PLOD3, p-PKCα, PKCδ, p-PKCδ, and β-actin. **f** Subcellular fraction analysis of R-H460 cells following siCON or siPLOD3 treatment. Proteins in each fraction were resolved by SDS-PAGE and immunoblotted with antibodies against PLOD3, PKCδ, lamin A/C, and α-tubulin. **g** R-H460 cells were transfected with 40 nM siCON or siPLOD3 and/or siPKCδ for 48 h. Cell death in R-H460 cells was determined by AV/PI staining (left). Protein levels of the indicated proteins were determined by western blotting (right). *P < 0.05. **h** R-H460 cells were transfected with 40 nM siCON or siPLOD3 and/or siPKCα for 48 h. Cell death in R-H460 cells was determined by AV/PI staining (left). Protein levels of the indicated proteins were determined by western blotting (right). *P < 0.05. **i** Protein levels of PLOD3, PKCδ, p-eIF2α, and p-IRE1 α were determined by western blotting. **j** Caspase-2, caspase-3, and caspase-4 activities in R-H460 cells (treated as in **b**) were determined by caspase activity assay. Data were collected using the Multiskan EX at 405 nm. *P < 0.05; **P < 0.01

It is well-known that PKCα or PKCδ is involved in the ER stress response, leading to apoptosis^29,30^. Moreover, PKCδ reportedly associates with and phosphorylates caspase-3 to promote apoptotic activity, after which activated caspase-3 cleaves PKCδ^31^. Thus, to investigate the relationship between PLOD3 and PKC, we compared the subcellular localization of these markers in R-H460 cells (Fig. 5a). The data showed that PLOD3 co-localized with PKCα and PKCδ in the nucleus and cytosol. Interestingly, PKCδ localized to the ER, similar to PLOD3. Next, as PLOD3 co-localized with PKCs, we evaluated the associated interactions in R-H460 cells. We immunoprecipitated exogenous PLOD3 from HA-PLOD3-, PKCα-, and GFP-PKCδ-transfected R-H460 cell lysates using a HA antibody and detected the immune complexes with HA, PKCα, and GFP antibodies (Fig. 5b, c). We observed an interaction between PLOD3 and PKCs in R-H460 cells. A proximity ligation assay confirmed that endogenous PLOD3 interacts with PKCs in vivo(Fig. 5d). A proximity ligation assay probe was used as a negative control. To examine whether PKC activity changes in the absence or presence of PLOD3, we transfected R-H460 cells with *PLOD3* siRNA. PKCα and PKCδ phosphorylation was remarkably increased by PLOD3 knockdown in R-H460 cells (Fig. 5e). These results suggest that PLOD3 regulates the activity of PKCα and PKCδ, leading to PKC-dependent apoptosis. Furthermore, we examined the localization of cleaved PKCδ, which is important for PKCδ-dependent apoptosis. Cleaved PKCδ was significantly induced in PLOD3-knockdown cells and was localized to the nucleus (Fig. 5f). Thus, we hypothesized that PKCs are related to PLOD3 depletion mediated-apoptosis in R-H460 cells. Accordingly, we investigated whether *PKCδ* siRNA blocks PLOD3 knockdown-induced subcellular events. R-H460 cells were treated with combinations of *PLOD3*, *PKCδ*, and control siRNA for 48 h and apoptosis was analyzed by AV/PI staining (Fig. 5g). *PLOD3* siRNA led to an increase in the apoptotic cell population (~39%); however, combined transfection with *PKCδ* siRNA significantly suppressed this increase by more than 14%. Immunoblotting results confirmed that cleaved PARP and active caspase-3 were remarkably decreased, without rescuing PLOD3 protein levels (Fig. 5g). Based on the results of interaction with PLOD3 and PKCα and increased PKCα phosphorylation by PLOD3 depletion, we examined whether *PKCα* siRNA blocks PLOD3 knockdown-induced apoptosis. To this end, R-H460 cells were treated with a combination of *PLOD3*, *PKCα*, and control siRNA for 48 h. Apoptosis was then analyzed by AV/PI staining and immunoblotting (Fig. 5h). *PLOD3* siRNA increased the apoptotic population (~41%), but combined transfection with *PKCα* siRNA suppressed this increase by more than 13%. Immunoblotting data confirmed that cleaved PARP and active caspase-3 were remarkably decreased, without rescuing PLOD3 protein level. Further, IRE1*α* and elF2a phosphorylation, as assessed by immunological analysis, was similar to that of cell death markers (Fig. 5i). Therefore, *PKCδ* siRNA inhibits the PLOD3-induced ER stress response. Next, we examined whether combined *PLOD3* and *PKCδ* siRNAs could affect caspase activity (Fig. 5j). *PLOD3* siRNA significantly increased caspase-2 activity by approximately 1.6-fold compared to control siRNA, but combined transfection with *PKCδ* siRNA further enhanced this increase by ~1.2-fold. Further, *PLOD3* siRNA significantly increased caspase-3 activity by ~2-fold compared to that with control siRNA, but combined transfection with *PKCδ* siRNA enhanced this increase by ~1.5-fold. Moreover, *PLOD3* siRNA significantly increased caspase-2 activity by ~1.5-fold compared to control siRNA, but combined transfection with *PKCδ* siRNA enhanced this increase by ~1.2-fold. Taken together, these results suggest that PKCδ, downstream of PLOD3, plays an important role in the PLOD3-mediated cell death mechanism.

### Loss of PLOD3 overcomes chemoresistance in vitro

**Fig. 6:**
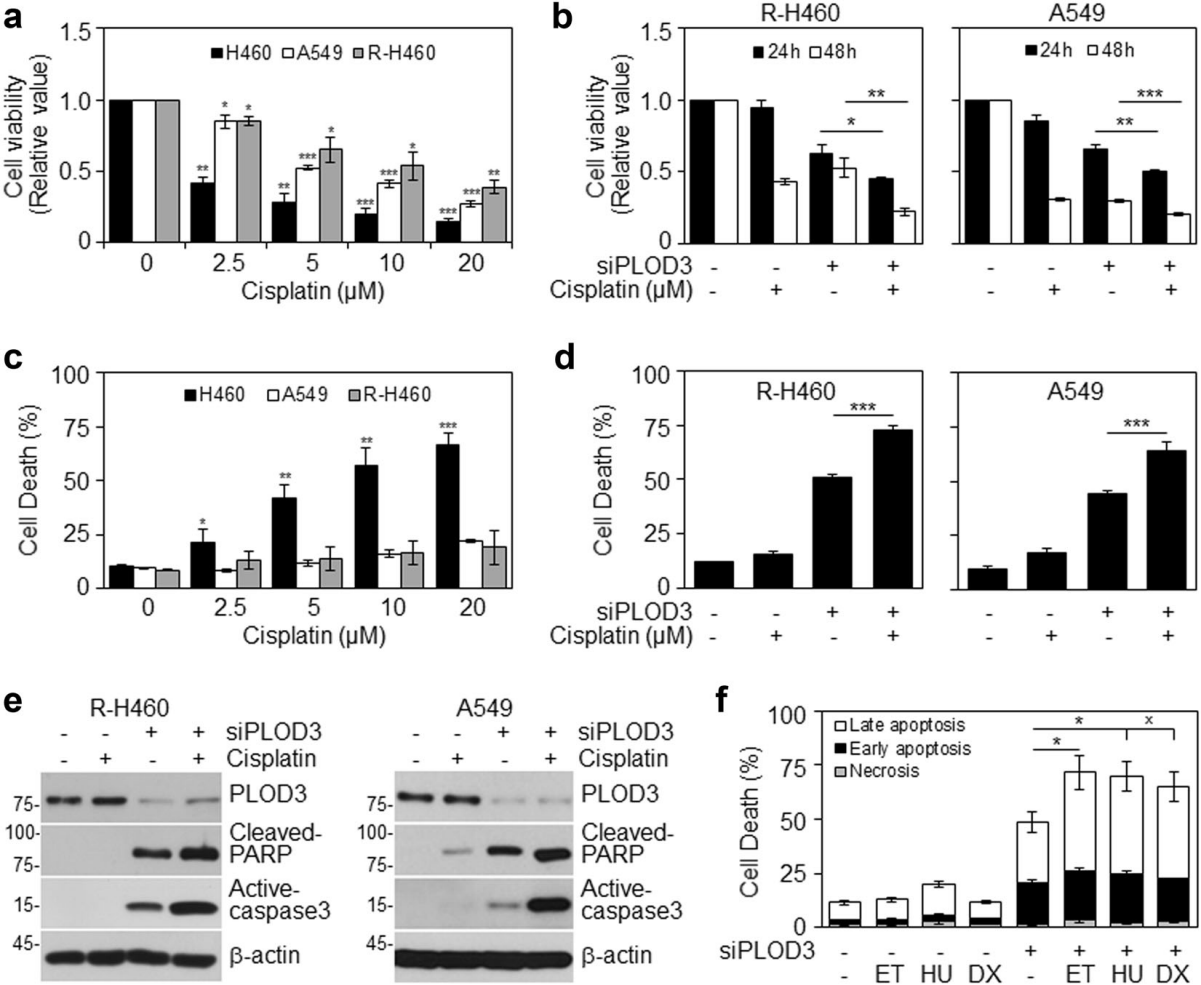
PLOD3 depletion overcomes chemoresistance* in vitro*. **a** Cells were treated with cisplatin for 48 h. Proliferation was detected by cell viability assays. *P < 0.05; **P < 0.01; ***P < 0.001. **b** A549 and R-H460 cells were transfected with 40 nM siCON or siPLOD3 for 24 h and then treated with cisplatin for another 48 h. The proliferation rate was determined by cell viability assays. *P < 0.05; **P < 0.01; ***P < 0.001. **c** Cisplatin-treated cells (for 48 h) were analyzed by FACS after AV/PI staining. *P < 0.05; **P < 0.01; ***P < 0.001. **d** Cell death (of cells treated as in **b**) as analyzed by AV/PI staining. ***P < 0.001. **e** Protein levels of cleaved-PARP and active-caspase-3 as determined by western blotting after *PLOD3* siRNA and cisplatin treatment. **f** R-H460 cells were transfected with siRNA in the absence or presence of 5 μM etoposide (ET) or 1 mM hydroxyurea (HU) or 0.1 μg/ml doxorubicin (DX). At 48 h after the drug treatments, the cells were analyzed by FACS after AV/PI staining. *P < 0.05

Next, we examined whether *PLOD3* status influences the chemosensitivity of cancer cells. Interestingly, radioresistant R-H460 cells were more resistant to cisplatin than their parental H460 cells, and resistance levels were similar to those of A549 cells (Fig. 6a, c). Next, we investigated the combined effect of cisplatin and *PLOD3* siRNA on the viability and cell death of R-H460 and A549 cells. The combinatorial treatment attenuated cell viability and increased cell death compared to cisplatin alone (Fig. 6b, d), suggesting that PLOD3 status might influence the chemosensitivity of cancer cells to cisplatin. Next, to investigate the effect of PLOD3 knockdown on apoptosis, we measured the expression of cleaved PARP and active caspase-3 by western blot analyzes (Fig. 6e). Compared to cisplatin alone, the combination of *PLOD3* siRNA and cisplatin resulted in significant increases in the levels of these two proteins in R-H460 and A549 cells. Whereas transfection of R-H460 cells with *PLOD3* siRNA alone induced approximately 47% cell death compared to 10% in controls, the co-stimulation of *PLOD3* siRNA-transfected cells with etoposide, hydroxyurea, or doxorubicin induced approximately 72%, 70%, and 65% cell death, respectively (Fig. 6f). Taken together, these results suggest that *PLOD3* status is strongly associated with cancer cell chemosensitivity.

## Discussion

PLOD3, which we identified as a radioresistance-related protein, has already been highlighted for its potential role in hepatocellular carcinoma, glioma, skin cancer, and prostate cancer^16–19^. However, for lung cancer, the specific mechanism of PLOD3 regulation in cell death has not been elucidated.

In the current study, *PLOD3* siRNA inhibited the proliferation of lung cancer cells and induced cell death, an effect that was augmented by radiation treatment, in cells and an in vivo model. We also found that (*a*) *PLOD3* is differentially expressed during tumor progression, (*b*) *PLOD3* expression promotes apoptosis in lung cancer by activating caspase-2,4-dependent signaling through ER stress-induced IRE1α activation and caspase-3-dependent apoptosis, and (*c*) *PLOD3* siRNA, combined with chemotherapeutic drugs, can increase cancer cell death, suggesting that PLOD3 is a potential therapeutic target in cancer. Thus, the present study provided the first evidence that PLOD3 inhibition can induce apoptosis in lung cancer cells, in addition to helping overcome radioresistance or chemoresistance.

Currently, PLOD2 is reportedly induced by hypoxia-inducible factor-1α under hypoxic conditions, which in turn enhances hypoxia-induced epithelial–mesenchymal transition in glioma^32^ and breast cancer cells^33^. Hypoxia-inducible factor-1α also regulates PLOD1 transcription in breast cancer; however, PLOD2 activity is more critical for HIF-1-induced cancer progression^33^. PLOD2 is also directly regulated by miR-26a-5p and miR-26b-5p, and PLOD2 expression is reportedly a possible prognostic marker for patients with bladder cancer^34^ and renal cell carcinoma^35^. Moreover, the E2Fs^36^ and FOXA1^37^ transcription factors have been demonstrated as regulators of PLOD2 during cancer progression. However, the regulation of PLOD1 and PLOD3 expression is not well understood. Thus, the upstream regulation of PLOD3 that could lead to the induction of cell death in our system remains to be elucidated. One report showed that miR-663a reduces PLOD3 biosynthesis by targeting the 3′-untranslated region of *PLOD3* mRNA, suggesting the interrelationship of this microRNA in regulating collagen IV secretion under physiological conditions and in response to ER stress. Consistent herewith, we focused on ER stress as an apoptosis-inducing factor as collagen is a major secretory protein in a variety of tissues, and defective secretion could result in physiological ER stress, fibrosis, and a number of disease states. As the pathway identified herein might represent a major physiological mechanism in lung cancer, it will be crucial to establish the role of the PKCδ-PLOD3-collagen axis in different tissues in future. Moreover, as PLOD3 is also present in the extracellular space, as well as in the serum and on the cellular surface^19^, our results suggest that PKCδ as a downstream effector of PLOD3 might also modulate other important extracellular PLOD3 functions^38^.

*PLOD3* mutations are reportedly associated with connective tissue disorders^11^. *PLOD3* knockout in embryos and cells was reported to be associated with reduced glycosylated hydroxylysines on type IV and VI collagen and abnormal distribution^39^. PLOD3 is overexpressed in hepatocellular carcinoma^40^ and is a potential diagnostic marker for early-stage disease^40^. Further, PLOD3 knockdown controls liver tumor incidence and growth rates in a spontaneous mouse model of hepatocellular carcinoma^40^. Our study showed consistent results for lung cancer and suggests that secreted PLOD3 could serve as a potential inducer of lung cancer metastasis or as a prognostic marker. We are currently expanding this mechanistic investigation in relation to metastasis using lung cancer cells and patient samples.

Interestingly, as we observed nuclear localization of cleaved PKCδ following PLOD3 knockdown, we hypothesized that *PLOD3* siRNA, via the production of ROS, might induce post-translational modifications in PKCδ, which allow its translocation to the nucleus. However, these events did not depend on ROS levels. Moreover, the hinge domain of PKCδ is the site for caspase-3 cleavage, which happens in the nucleus and laeds to the release of the δ-catalytic fragment (δCF), corresponding to the kinase domain. Thus, in some systems, apoptotic cell death is associated with caspase 3-dependent PKCδ cleavage; the formation of the catalytically active fragment of PKCδ in our cellular model following *PLOD3* siRNA treatment should be explored further in future studies.

Recently, diverse molecular biomarkers of drug resistance have been identified. For example, the protein disulfide isomerases PDIA4 and PDIA6 regulate resistance to cisplatin-induced apoptosis in lung adenocarcinoma^41^. The copper transporter CTR1 is involved in the uptake of cisplatin and has been developed as a therapeutic target^42^. Thus, medical protocols should be planed according to the character of each patient and it needs to find more efficient biomarkers for identifying the molecular mechanisms of cisplatin resistance. Our results suggest that chemotherapy could be more effective in combination with RNAi-mediated knockdown of *PLOD3*. Certainly, for the improvement of such a therapeutic strategy for clinical application, a suitable vector system is necessary. We also need to explore the effects of *PLOD3* knockdown on normal tissues in future before clinical application of this strategy can be contemplated. We demonstrated that the level of activated PKCδ or PKCα was increased upon treatment with siPLOD3 and that the siPLOD3-induced apoptosis was decreased by inhibiting the expression of PLOD3 and PKCδ or PKCα. Thus, we are further planning to identify the functional relationship between PLOD3 and PKCδ or PKCα; the cellular events or functional mechanisms underlying these also need to be elucidated.

In summary, the PLOD3 siRNA used in this study are potent tools to modulate *PLOD3* expression and they might ultimately be developed into attractive anti-tumor therapeutics. Further, PLOD3 might represent a prognostic biomarker and a target for reversing cisplatin resistance in lung cancer.

## Materials and methods

### Cell culture and treatment

Human lung cancer cell lines (H460 and A549) were purchased from ATCC (Manassas, Virginia, USA), and we established a radioresistant H460 (R-H460) cell line derived from parental radiosensitive H460 lung cancer cells treated cumulatively with 2 Gy radiation twice a week for 20 weeks^5^. H460, A549, and R-H460 cells were cultured in RPMI-1640 medium supplemented with 10% fetal bovine serum. Cells were irradiated using a 137 Cs-ray source (Atomic Energy of Canada) at a dose rate of 3.81 Gy/min. Where indicated, cells were treated with etoposide, hydroxyurea, doxorubicin, and cisplatin to induce apoptosis. Caspase inhibitors [Z-VAD-FMK (Adipogen, Z-VDVAD-FMK, Z-DEVD-FMK, and Z-YVAD-FMK (R&D Systems)] were used to block caspase activation. Inhibitor APY29 was used to block IRE1α activation. Where indicated, cells were treated with 3-methyladenine to block autophagy.

### PLOD3 siRNA transfections

The following human PLOD3-specific siRNAs, synthesized by Genolution, were used: #1; 5ʹ-GGUUAAAGAAGGAAAUGGAUU-3ʹ, #2; 5ʹ-GGAAGUACAAGGAUGAUGAUGACGACGA-3ʹ, #3; 5ʹ-AUAUGAUCAUCAUGUUUGUUU-3ʹ, #4; 5ʹ-GCCUUAAUCUGGAUCAUAAUU-3ʹ. The siRNA duplexes were transfected into cells using Lipofectamine^®^ RNAiMAX Reagent according to the manufacturer’s guidelines.

### Western blot analysis

Western blot analyzes were performed as described previously^16^ using primary antibodies targeting the following proteins: PLOD3 (Proteintech Group, Chicago, IL, USA); p-PERK, PERK, p-eIF2α, HA, GFP, lamin A/C, α-tubulin, PKCα, PKCδ (Santa Cruz Biotechnology); cleaved-PARP (Asp214), cleaved caspase-3, eIF2α, IRE1α, γH2AX, and p-PKCδ (Cell Signaling Technology); p-IRE1α (Novous); ATF6, caspase-2 and caspase-4 (Abcam); and β-actin (Sigma) was used as a loading control.

### Quantitative reverse transcription-polymerase chain reaction (qRT-PCR)

Total RNA was isolated using an RNeasy Mini kit (Qiagen). qPCR was performed in triplicate using a PIKOREAL 96 (Thermo) and SYBR Premix Ex Taq (Takara Bio, Shiga, Japan). A two-temperature thermocycling program was used, with 42 cycles of 95 °C (denaturation) and 55 °C (annealing). Target-gene amplification signal was normalized to that of *GAPDH* in the same reaction.

### Cell viability assay

Cells were seeded at 5000 cells/well in a 96-well plate and incubated for 24 h in accordance with the indicated experimental conditions. For quantification of cell viability, an equal volume of culture medium containing Cyto X^TM^ Reagent (LPS solution) was added to the cells and the plate was incubated for 4 h. Cell viability was then measured using Multiskan EX (Thermo Fisher Scientific, Germany) at 450 nm.

### Fluorescence-activated cell sorting (FACS)

Cells (3 × 10^5^) were seeded in a 60-mm dish and exposed to the indicated experimental conditions. For quantification of cell death, cells were trypsinized, washed in phosphate-buffered saline (PBS), and then resuspended in 1× binding buffer. Cells were incubated with annexin V (AV) and propidium iodide (PI) for 15 min, and analyzed with a FACScan flow cytometer (Becton Dickinson).

### Clonogenic assay

Cells were seeded in triplicate into 60-mm tissue culture dishes at 2, 4, 8, 16, and 32 × 10^2^ cells/dish and exposed to 0, 2, 4, 6, and 8 Gy, respectively. Cells were exposed once to different doses of radiation. After 14 days, colonies arising from surviving cells were stained with trypan blue and counted using a colony counter (Imaging Products).

### Tumor xenograft animal model

Studies with the tumor xenograft animal model were performed as described previously^43^ using control siRNA or PLOD3 siRNA #2 (KIRAMS 2015–0070).

### Immunofluorescence confocal microscopy

Immunofluorescence staining was performed as described previously^43^ using primary antibodies against PLOD3 (Proteintech), PKCα, PKCδ, and GRP94 (Santa Cruz Biotechnology). Cell nuclei were counter-stained with DAPI. Images were acquired using a confocal laser-scanning microscope (model LSM 710, Carl Zeiss) and were processed with ZEN 2009 Light Edition (Carl Zeiss).

### Data mining using Kaplan–Meier plotter

Kaplan–Meier survival curves in relation to *PLOD3* expression were generated for lung cancer, using KM Plotter (http://kmplot.com/). Cancer type was defined as lung cancer and data type as mRNA, whereas analysis type was defined as cancer vs. normal.

### Caspase activity assay

Caspase activities were measured using caspase family activity assay kits (Abcam) according to the manufacturer’s recommendations. Data were collected using a Multiskan EX at 405 nm.

### Proteasome activity

Proteasome activities were measured using proteasome-Glo^TM^ chymotrypsin-like, trypsin-like, and caspase-like cell-based assays (Promega) according to the manufacturer’s recommendations. Data were collected using a Victor X2 multi-label reader (Perkin Elmer).

### ROS assay

R-H460 cells were treated with siRNA under the indicated experimental conditions. The cells were incubated with 10 nM 2′,7'-dichlorofluorescein diacetate (Molecular Probes) in the dark at 37 °C for 30 min. Cell staining was examined with a laser-scanning confocal microscope (model LSM 710, Carl Zeiss) equipped with an argon laser tuned to an excitation wavelength of 488 nm, LP505 emission filter (515–540 nm), and Zeiss Axiovert -100X objective lens. Two groups of cells were randomly selected from each sample.

### In situ proximity ligation assay

Paraformaldehyde-fixed R-H460 cells were permeabilized with 0.2% Triton X-100, washed, and blocked with blocking solution (Olink Bioscience). Mouse monoclonal anti-PLOD3 antibody (Proteintech Group) together with rabbit polyclonal antibodies against PKCα, PKCβI, PKCβII, PKCγ, PKCδ, and PKCζ (Santa Cruz Biotechnology) were used for the proximity ligation reaction. The assay was conducted using the Duolink Detection Kit (Sigma) according to the manufacturer’s protocol.

### Plasmid construction and transfection

Plasmids were constructed by standard cloning techniques and were verified by DNA sequencing. Human PLOD3 cDNA (wild type) purchased from Origene (Cat. No. SC324563) was PCR-amplified and cloned into the pcDNA3.1 vector. Wild type PLOD3 was amplified with primers 5′-GATGGATCCATGACCTCCTCGGGGCCTGGA-3′ (BamHI) and 5′-TCTCGAGTCAGGGGTCGACAAAGGAC-3′ (XhoI), and the amplicon was inserted between BamHI and XhoI in the pHA vector. PKCα and GFP-PKCδ expression vectors were purchased from the Korea Human Gene Bank. Plasmids were transfected using Mirus-2020 Reagent according to the manufacturer’s guidelines.

### Immunoprecipitation

R-H460 cells were transfected with vectors under the indicated experimental conditions for 48 h. The cells were washed twice with PBS, harvested, and lysed for 30 min in NP-40 buffer [50 mM Tris-HCl (pH 8), 150 mM NaCl, 1% NP-40, and 100X protease and phosphatase inhibitor cocktail]. Samples were diluted to 500 µg of protein in 800 µl of buffer and pre-cleared for 1 h at 4 °C with 50 µl of a 50% slurry of protein A/G-Sepharose beads (GE Healthcare). After brief centrifugation to remove pre-cleared beads, 1 µg of antibody against HA (Santa Cruz Biotechnology) was added to each sample and incubated on a rocking platform at 4 °C overnight. The immune complex was precipitated by incubation with 40 µl of protein A/G-Sepharose beads at 4 °C for 4 h. The beads were washed thrice with immunoprecipitation buffer and then boiled with sample buffer [0.1 M Tris-HCl (pH 6.8), 4% SDS, 40 mM EDTA, 20% glycerol, and β-mercaptoethanol].

### Subcellular fractionation

Cells were lysed using subcellular fractionation buffer (250 mM sucrose, 20 mM HEPES (pH 7.4), 10 mM KCl, 1.5 mM MgCl_2_, 1 mM EDTA, 1 mM EGTA, 1 mM DTT, and protease inhibitors), and the lysate was passed through a 25-gauge needle 10 times using a 1-ml syringe. The lysate was placed on ice for 20 min and centrifuged at 3000 rpm for 5 min to obtain nuclear pellet. The pellet was washed with fractionation buffer 11 times, and the supernatant was centrifuged at 8000 rpm. The supernatant here refers to the cytosolic and membrane fraction.

### Statistical analysis

Cell culture experiments were performed at least in triplicate. All data are expressed as mean ± standard deviation values. Statistical differences between groups were assessed using Student’s *t*-test (two-tailed) analysis. *P*-values were interpreted as follows: not significant (n.s.), *P < 0.05, **P < 0.01, and ***P < 0.001.

## Supplementary information

Supplementary fig1

Supplementary fig2

Supplementary fig3

Supplementary fig legend

Supp. Table
