# Supplementary figures and images for: PLOD3 suppression exerts an anti-tumor effect on human lung cancer cells by modulating the PKC-delta signaling pathway
Source: Cell Death Dis. 2019 Feb 15;10(3):156. doi: 10.1038/s41419-019-1405-8 (PMC6377650; doi:10.1038/s41419-019-1405-8)

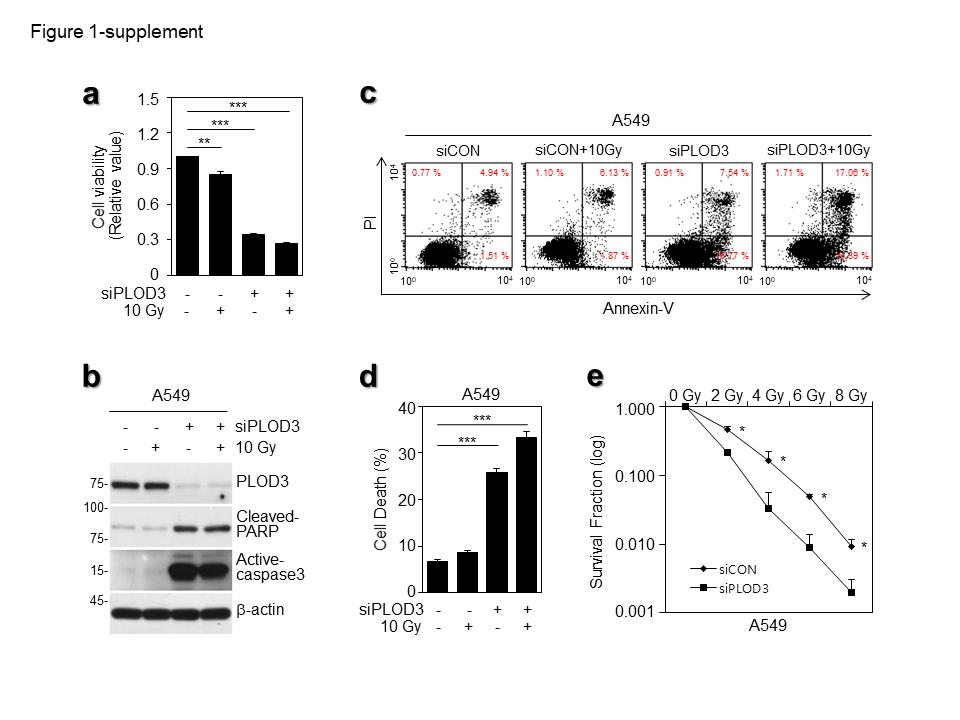

Supplement: Supplementary file 1 — Supplementary fig1 [file 41419_2019_1405_MOESM1_ESM.tif]

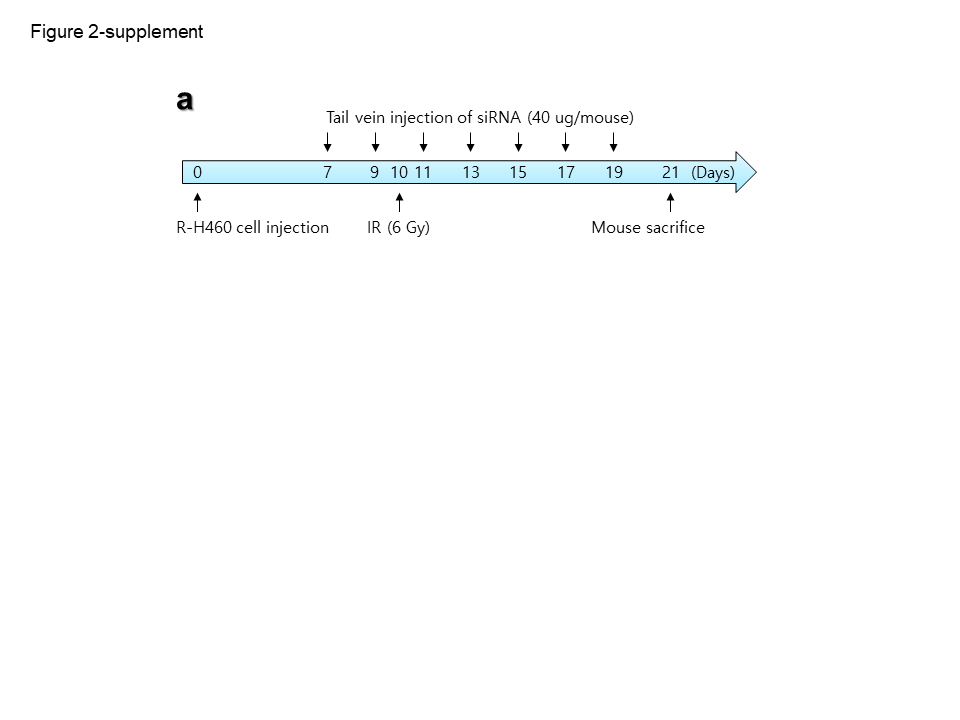

Supplement: Supplementary file 2 — Supplementary fig2 [file 41419_2019_1405_MOESM2_ESM.tif]

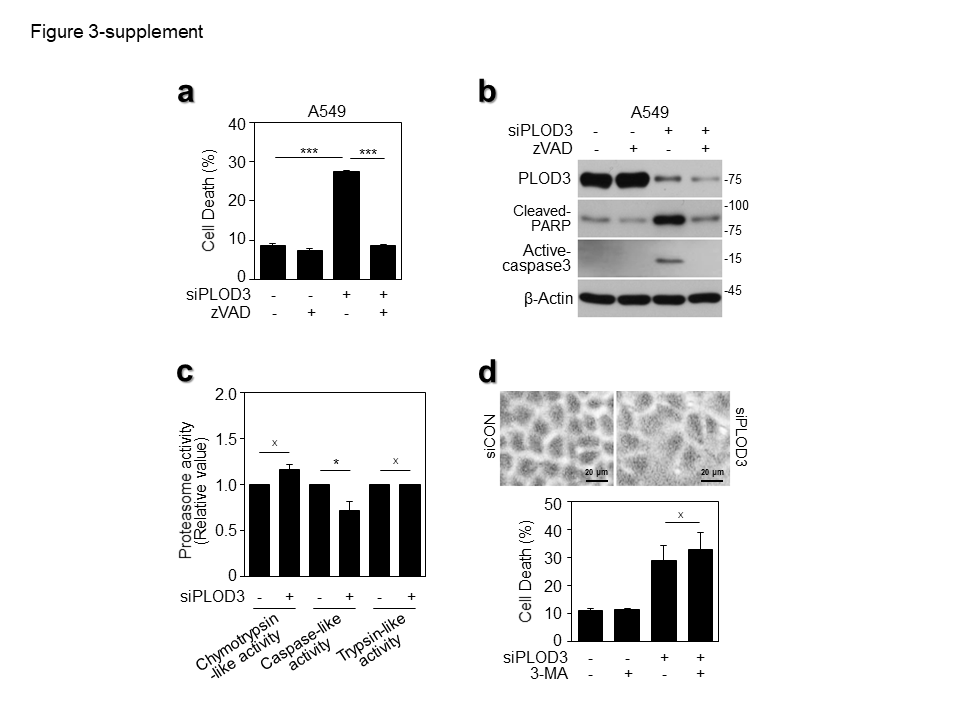

Supplement: Supplementary file 3 — Supplementary fig3 [file 41419_2019_1405_MOESM3_ESM.tif]
