# Supplementary material for: PLOD3 suppression exerts an anti-tumor effect on human lung cancer cells by modulating the PKC-delta signaling pathway
Source: Cell Death Dis. 2019 Feb 15;10(3):156. doi: 10.1038/s41419-019-1405-8 (PMC6377650; doi:10.1038/s41419-019-1405-8)
Supplement: Supplementary file 4 — Supplementary fig legend [file 41419_2019_1405_MOESM4_ESM.docx]

**Supplementary Figure Legends**

**Fig1. Down-regulation of PLOD3 decreases radioresistance in A549 cells.** (a) Analysis of cell viability in A549 cells untreated or treated with 10Gy radiation after transfection with 40 nM siCON or siPLOD3. ***P* < 0.01; ****P* < 0.001 (b) Protein levels of PLOD3, cleaved-PARP and active-caspase-3 were determined by Western blotting. (c,d) A549 cell was treated with 10 Gy radiation for 48 h after siPLOD3 transfection. Determination of cell death in A549 cells treated as in b by the Annexin V/PI staining for FACS analysis. ****P* < 0.001 (e) Survival fraction of A549 cells treated with a single radiation (0-8 Gy) after transfection with 40 nM siCON or siPLOD3, measured 2 weeks after radiation treatment. **P* < 0.05.

**Fig2. Loss of PLOD3 promotes radiosensitivity *in vivo*.** (a) Schematics of the experimental design to assess the effect of PLOD3 knock-down with irradiation on tumorigenicity by subcutaneous injection with 1 X 10^6^ R-H460 cells. 7 days after the injection of R-H460 cells, the mice were subjected to tail vein injection every 2 days with siCON or siPLOD3 (40 μg siRNA/mouse). 10 days after the injection of R-H460 cells, 6Gy X-ray radiation to R-H460 xenograft tumors.

**Fig3. PLOD3 depletion induced cell death is independent to autophagy and proteasome activity.** (a) Cell death analysis of A549 cells left untreated or treated with 100 µM Z-VAD-FMK (pan-caspase inhibitor) after transfection with siPLOD3, measured 48 h after treatment. ****P* < 0.001 (b) Protein levels of cleaved PARP and active-caspase-3 determined by Western blotting. (c) Measurement of the activity of proteasome captures from R-H460 cells expressing siPLOD3. Captured proteasome are incubated in the presence of commercially available fluorogenic substrates to measure their chymotrypsin-like, caspase-like and trypsin-like activities, respectively. **P* < 0.05 (d) R-H460 cells were photographed under Nikon Eclipse Ts2R-FL after siPLOD3 treatment (upper). Cells were treated with 3-MA in the presence or absence of siPLOD3 for 48 h, and the cell death rate was measured by performing FACS analysis (lower).
