# Supplementary material for: PLOD3 suppression exerts an anti-tumor effect on human lung cancer cells by modulating the PKC-delta signaling pathway
Source: Cell Death Dis. 2019 Feb 15;10(3):156. doi: 10.1038/s41419-019-1405-8 (PMC6377650; doi:10.1038/s41419-019-1405-8)
Supplement: Supplementary file 5 — Supp. Table [file 41419_2019_1405_MOESM5_ESM.docx]

**Supplementary table 1**. List of primer sequences for qRT-PCR.

| Gene | sense/antisense | Primer sequences |
| --- | --- | --- |
| *PLOD3* | sense | 5ʹ-GCG CCA GTG GAA GTA CAA GGA T-3ʹ |
|  | antisense | 5ʹ-CAC TTC ATC TAA AGC CCC GTT GA-3ʹ |
| *spliced*  *XBP-1* | sense | 5ʹ-CTG AGT CCG CAG CAG GTG-3ʹ |
|  | antisense | 5ʹ-ATC CAT GGG GAG ATG TTC TGG-3ʹ |
| *unspliced XBP-1* | sense | 5ʹ-CAG CAC TCA GAC TAC GTG CA-3ʹ |
|  | antisense | 5ʹ-ATC CAT GGG GAG ATG TTC TGG-3ʹ |
| *GAPDH* | sense | 5ʹ-CAT CTC TGC CCC CTC TGC TGA-3ʹ |
|  | antisense | 5'-GGA TGA CCT TGC CCA CAG CCT-3ʹ |
